# Illustrations of Difficult Parturition

**Published:** 1859-09

**Authors:** 


ART. III.—*Illustrations of Difficult Parturition.* By JOHN HALL DAVIS, M.D., Lecturer on Obstetric Medicine, etc. etc. pp. 284. London: Churchill. 1858.

THIS little work belongs to a class always hailed with pleasure by the practitioner, because read with so much profit. Containing the records of difficult cases which have occurred in an extensive practice, with the remarks, upon doubtful points, of one whose learning has been tempered by experience, the older practitioner finds in them a counterpart of the difficulties he has himself encountered, and the younger the nearest possible approach to personal experiences.

The author is a man of high standing in this branch of practice, a mantle of great professional reputation having descended upon him from his father; he has enjoyed for years the advantages to be derived from several appointments to public institutions, and he has now a large metropolitan practice; his learning, his experience, and his position are, therefore, guarantees that he would not occupy the time of his professional brethren in vain, nor call upon them for a hearing without having something to communicate worthy of their attention.

The work is, as its title indicates, a clinical record of cases, and we are not, of course, to expect that completeness which characterizes a treatise, nor even the exhaustion of those subjects which are considered. About two-thirds of its pages are occupied with the history of cases, one hundred and forty-four in number, and, so far as these are concerned, there is no need or room for criticism; the other third contains a brief exposition of the principles which guide the author in resorting to instrumental assistance in conducting the different classes of labor, and in remedying the various forms of difficulty. This part of the work is excellent; it bears the marks of experience and study; and the remarks upon the treatment of the various forms of difficult parturition exhibit so much sound judgment in regard to the choice of means, and inculcate so great caution as to their use, as to render them valuable to every member of the profession, but more especially to the young. The work closes with the statistics and analyses of seven thousand three hundred and two deliveries which have taken place under the author's superintendence.

The introductory chapter contains a brief sketch of the causes of delayed and difficult labor as depending upon the position of the child, the forces which propel it, or the passages through which it is transmitted, and a very general notice of the treatment required for the different kinds and degrees of difficulty. The remedial measures for difficult parturition, of course, vary in character as widely as do the nature of the cases which render assistance necessary, and nowhere is skill and sound judgment more requisite on the part of the practitioner than in discovering the cause of the difficulty and in adapting remedies to it; his success will be marked in proportion to his acuteness in observation, his judgment in selection, and his promptitude in administration. Some of the rarer causes of delay are alluded to in this introductory chapter, such as irritating ingesta, which are stated to interfere sometimes with the progress of labor, and even to occasion convulsions; cases of this kind are mentioned, and find their appropriate treatment in an emetic. Especial pains are taken by the author to impress the fact that the bladder cannot be too carefully watched during labor; a distended condition of this organ is frequently the cause of vexatious delay which a timely resort to the catheter will remove, while inattention to this point has been followed by the most serious results. In some cases, no cause for inefficient uterine action can be found but general debility; here light nourishment and mild cordials form the appropriate treatment. When the uterus is sluggish in exerting its powers, the administration of ergot is counseled; "obstructing membranes must be divided," while "cicatrixes, and smallness of the vagina, not arising from any morbid source," may generally be trusted to the natural efforts, although they sometimes require the administration of relaxing remedies. In those cases where delay is caused by rigidity of the soft parts, "sometimes the cause, sometimes the result, of premature discharge of the waters," the rigidity may be removed by tartar emetic, or, when the strength permits, by blood-letting, or by the use of opium. The latter remedy is to be used with caution, and is sometimes most successful when exhibited after moderate depletion. It is to the treatment of this condition of things that chloroform has been especially recommended; we think, however, that its friends have claimed too much certainty for its action in removing rigidity of the soft parts, for we have seen instances where it failed, like the other remedies, in overcoming the cause of delay, so annoying to the accoucheur and so painful to the patient; and Dr. Davis intimates as much.

"For the purpose of quieting muscular spasms, soothing the nervous system, and so relaxing these rigid conditions of the genital passage, *chloroform* in later years has been much resorted to by some practitioners; and I must confess that whenever I have employed it with this intention, I have rarely found it fail me. But it is, in my opinion, an agent of too great potency to be used without great caution."

The concluding sentence of this paragraph gives the author's position in regard to the use of anæsthetics in obstetrical practice. He uses them himself whenever necessary; he bears testimony to their beneficial effects, yet evidently is afraid that upon the strength of his recommendation others may do mischief with them. This extreme caution in regard to the use of anæsthetics, and of chloroform in particular, will not be surprising to those acquainted with the general feeling which has prevailed against these agents, ever since their introduction, in the city where the author resides, and that one who uses chloroform and counsels its administration in obstetrical practice there, has against him the authority of one of the most eminent accoucheurs and teachers of the metropolis, who is not only strongly, but bitterly opposed to its use. It may be that some of the extreme caution manifested by the author speaking of this article may be thus accounted for, but whether we are right in our conjecture or not, we cannot help expressing our regret that he does not occupy a more advanced position in regard to the use of one of our most valuable remedies.

While upon this subject we will conclude it by quoting some other paragraphs in which the remedy is mentioned, and let the author speak for himself. The first we take from the directions for the application of the forceps.

"In some cases there is extreme restlessness, and our patient is perfectly uncontrollable. Here, the risks of a forceps operation would be greatly increased, unless we adopted means to prevent them; we may, therefore, find it expedient to resort to the sedative influence of chloroform I may observe, in passing, that I do not often appeal to this agent in ordinary labor, and by no means in all obstetric operations; but it seems to me that this is one of the conditions in which this anæsthetic vapor may be usefully employed."

The other paragraph is from the directions for the management of those fearfully trying cases where the presentation is transverse, the waters have long escaped, and the uterus is firmly contracted around the child.

"As regards the last agent, [chloroform,] it certainly removes all suffering, and, in most cases, produces the necessary relaxation. I have known it fail, however, more than once, in effecting that object, although the patient was reduced to perfect unconsciousness. Moreover, there are constitutions and conditions of the thoracic organs in which it cannot be safely administered; and, in some instances, it has appeared to me to have predisposed to hemorrhage after delivery. The uterus has been left in a state of inertia, considerable trouble and anxiety have been occasioned, although the patients in the end did well."

By far the most important point in cases of difficult parturition is to determine when interference is necessary, to decide that the patient has reached a point beyond which she cannot go without running serious risk. To the young practitioner, a decision upon this point is peculiarly embarrassing, and is only equaled in its difficulty by its importance. We are told by the author that we must regulate our conduct as to interference by "*time* and *symptoms.*" To the first part of this proposition we are inclined to object entirely, and practically the author is with us, although theoretically he lays down the rule as above; there is always a saving clause introduced which throws the element of time, just where it should be, into a very subordinate position as bearing upon this point. The different limits which have been assigned to our delay by different authorities, varying from a period of four to twenty-four hours, and the fact that cases are given by the author as well as by other eminent obstetrical writers, in which the most serious results have followed but a very short period of delay, are sufficient to show that we cannot decide this question by a mere reference to the time which has elapsed since the labor began. We give, however, the author's rule.

"In my own practice I adopt as a rule, modified by the circumstances of each case, a limit of from four to six hours of arrest of the head in the second stage of labor, under strong pains, in one position, from which it neither advances nor retires."

In making these remarks we do not overlook the fact that the author is speaking of a class of cases "where difficulty arises with a pelvis of apparently standard measurements, and where nothing positive can be ascertained about the cause of the impediment." Yet even here there are so wide variations in the energy, frequency, and force of the uterine contractions, and in the constitution, temperament, and state of health of the patient, as to render it impossible to adopt the rule of the author, or any other course of action based upon time alone; and we believe the prominence given to the ground of decision arises more from obedience to the custom of writers than from any conviction of its value derived either from theory or from practice.

The symptoms which should excite anxiety for our patient's safety, and indicate that interference is necessary, are then detailed; they are "a feverish pulse and skin, loaded tongue, dry, heated vaginal mucous membrane, a brown, often offensive discharge from the uterus, tenderness and swelling of the genital surfaces, and soreness of the abdomen."

"As an aid to our decision in favor of interference, I may refer to a sign, which I have for several years past found useful as an intimation of approaching danger from protracted pressure in obstructed labor, namely, an olive-colored or brownish slimy discharge, a depraved secretion from the mucous membrane, the result of long-continued irritation. The child may often be saved after the occurrence of this discharge, which differs in character from that of meconium."

A sketch of the mechanism of labor precedes the consideration of operative deliveries; in this the author follows Nægele, and there is nothing different from what is usually found in text-books; the two transverse positions, in which the face is directed to the right or to the left ilium, the first and second presentations of Ramsbotham, are called "irregular," and stated to occur very rarely. The particulars of the mechanism of each position are briefly given, and the importance of fully understanding them impressed by the following lines:—

"The practitioner who is well acquainted with the above positions, and the mechanism of the head's advance in labor, will not be so ready to withhold his reliance on nature, as he would be, and as our forefathers were, without the advantage of that knowledge"

In the chapter upon forceps deliveries, the indications for their use are given, and the precautions to be observed in their application, as well as the characteristics of a good instrument. In regard to the latter point, the author differs from the majority of British authorities in preferring one with the pelvic curve; the instrument he prefers was introduced by his father, and is known as "Davis's forceps," its chief peculiarities being the width of the fenestræ of the blades and the great convexity of their outer edge as compared with the concavity of their inner. This instrument is fully described and figured in Professor Meigs's work on obstetrics; he also gives this instrument the preference, and dwells on its peculiar excellences, perfect adaption to the curve of the pelvis, and the great equality with which the pressure upon the child's head is distributed. In counseling the use of the forceps, as well all other instruments, the author inculcates the greatest circumspection and caution.

"In the use of the forceps we must be most gentle; no hasty nor violent manœuvres are admissible, either in the introduction of the blades or in the traction which we employ. We must also be careful as to the *degree and duration* of extractive force which we exert; serious have been the results of inattention to this precaution. Most scrupulous must we be as to the safety of the mother in every way, knowing that in one short moment, by one rash act, we may inflict upon her an irreparable, nay, a fatal injury."

The author does not believe cases very frequently occur which justify the application of the forceps when the head is at the brim of the pelvis, the difficulties being of such a nature when the head is arrested so high up, and the long forceps being so dangerous an instrument, as to call for the safer operation of craniotomy. In regard to the well-known practice of Simpson in cases of this description, he gives the following opinion:—

"It has been proposed by Professor Simpson, in revival of a practice of bygone times, to substitute turning for long forceps and craniotomy operations, in all cases of contraction of the pelvic brim, where the passage of the hand is at all possible. But it appears to me that the results to the child in footling cases, *ab origine*, and in those derived from version in cross presentations, even in standard pelves, are not calculated very favorably to impress one in its favor. We have to reflect, also, upon the additional suffering and risk which the operation of turning might occasion to the mother, and with so little prospect of an equivalent good.

"I am, nevertheless, disposed to admit, that it may be right to attempt the operation of turning in a small pelvis, in *extremely rare instances*."

While he distinctly teaches the great danger likely to result from the use of the long forceps, he cannot find, in turning, any substitute for them in the cases for which they are recommended, but fully indorses the opinion of Denman, that turning, under the circumstances, "is rather to be considered among those things of which an experienced man may sometimes avail himself in critical situations, than as submitting to the ordinary rules of practice."

A few pages of interesting statistics are given, showing the frequency of forceps and craniotomy deliveries in British and continental practice; and this subject is then closed with evident, and allowable, self-gratulation upon the fact that it has been the author's "good fortune never to have lost a single patient after forceps delivery." This success he attributes to caution in the employment of the instrument and careful after-treatment of the patients. Many of the cases bear testimony to extreme watchfulness of the earliest indications of inflammation after instrumental delivery, with prompt and decided treatment, and to a careful practical attention to all the admirable directions laid down in the remarks upon "treatment after operative delivery." Every reader will look upon the after-treatment as no mean element of the success which has thus far attended the author, although he bears willing testimony to the fact that his professional friends who have called him in consultation "have in no instance added the dangers of delay to the difficulties before them."

In considering the cases which require the operation of craniotomy, the reader is again warned against injudicious and too long-continued efforts to deliver with the long forceps, as being fraught with danger to the mother, and likely, therefore, to result in the loss of both instead of one; he gives cases which justify his remarks upon the subject, and states that "in a pelvis distorted at the conjugate diameter to the extent of two inches and a half, a prior use of the forceps, upon a child at or near full time, would be most improper." He gives the space necessary for delivery by the natural passages, with "every attainable advantage from operative skill, and the best adapted instruments," as one and a half inches in the antero-posterior direction at the brim, or of "at least one and three-eighths of an inch in the conjugate, and three and a half inches from ilium to ilium."

In the beginning of the consideration of this subject he plainly states the principle that although craniotomy may have been necessarily performed in one labor, the patient may be delivered without instruments and without difficulty the next time; and, on the other hand, that because a woman has been once delivered without the operation, it is no proof that it may not be necessary at her next confinement. These paragraphs may prove of consolation to those who have been obliged to resort to the operation where the patient had formerly borne children; in these cases the public are too apt to take the natural delivery as the standard of success, and unjustly to conclude that because a woman has once given birth to a child without assistance she can do so again. We have known two cases in which craniotomy was considered necessary by several experienced practitioners, in both of which the patients had previously, although with great difficulty, given birth to a living child. We have a patient now under our observation whom we have attended in these labors; the first two were each of more than thirty hours' duration, and would have been terminated with the forceps, had there been any instruments within a day's journey, and both children were still-born; yet in her third confinement she was delivered of a living child within one and a half hours after the first pain was felt. The first children were males, the last one a female, and their different size would fully account for the different character of the labors. Inordinate ossification of the head, as the author remarks, may also render craniotomy necessary in a case where the patient has borne living children before, and differences in other respects make it impossible to judge of one labor by another.

The details of the operation of craniotomy bring to notice the instruments which the author uses. The perforating scissors of Smellie, for opening the cranium, he adopts with authorities generally; but the crotchet, he says, he has "long discarded, after ample trial, as a most inefficient instrument in the more difficult cases." He has given two or three cases in illustration of the statement that he has been called upon repeatedly to finish deliveries which had been commenced with the crotchet, "in the hands of practitioners not otherwise undexterous." He, therefore, prefers the craniotomy forceps as meeting the demands of any difficulty, and gives the following description of what he considers to be the proper construction:—

"In this form there project from one blade, which is passed *within* the skull, three strong teeth disposed in a triangular form, which, when the blades are locked, fit into three corresponding openings in the opposite or outside blade, but not passing through, are guarded from doing injury to the mother. The blades are separable, and the lock and handles made like those of the English forceps"

We have been thus particular to give a description of the instrument in the author's own words that our remarks upon this part of the subject may be better understood. We do not claim sufficient experience to give any great weight to our judgment upon the comparative merits of these two instruments, but we have had sufficient to convince us that both of them are generally faulty in their construction. That the author has been influenced in his decision by the bad work of the instrument-makers we do not for a moment suppose; but, for ourselves, we hesitate to condemn an instrument which we have not seen of proper shape and construction. The crotchet recommended by all modern writers has a curved shaft, to allow of easy and efficacious application to the head as it lies in the pelvis, and of a proper expenditure of the force exerted. The instrument is thus figured, with a gentle curve from the handle to the turn of the hook, on page 356 of Churchill's Theory and Practice of Midwifery,* and in plate xvii. fig. 5 of the same author's Researches in Operative Midwifery; and in plate xv. fig. 8 of the same work, is represented Mesward's crotchet, of which the modern instrument is a modification, and in which the curve of the shaft is much more strongly marked. Yet a crotchet in our possession, made by one of the best-known instrument-makers of this country, is perfectly straight from the handle to the bend of the hook, and the point of the latter is bent down so nearly parallel to the shaft as to render it almost impossible to obtain a hold or exert any force with it. An examination of the cuts we have mentioned,† and a moment's reflection, will convince any one not only of the difficulty of applying such an instrument, but of the fact stated by Murphy,‡ that the force exerted by it is thrown entirely on the point of the instrument, and tends rather to break off pieces from the skull than to draw forward the head of the child. Murphy is, we believe, the only writer who dwells upon the importance of a properly-shaped instrument, and upon the usefulness of such a one as we have described, and which is a pattern, so far as we have seen, of those in the hands of practitioners in this region, certainly of all we have seen from the same maker.

Again, to refer to the craniotomy forceps. So far as our opinion goes, we are ready to assent to the author's opinion as to the great superiority of this instrument over the crotchet. The instrument we have used resembles the one described above, except that the blades are not separable, but are connected by a permanent joint. Were this the only objection, there would be but little ground for complaint, but the projecting teeth, instead of being placed upon the blade made to pass inside the skull, are placed upon the one curved to pass between the pelvic wall and the head! The difficulty of using such an instrument may be imagined. In two cases, after having succeeded in its application, it proved a most effective means of accomplishing the delivery, which the crotchet had failed to do; but in a third case it was impossible to apply it, and the fatal result which followed we attribute to the delay occasioned by the faulty construction of this instrument.

We have taken up so much space already that none is left for the further consideration of this, or other subjects. There are chapters upon the indication of premature labor, upon breech, footling, and transverse presentations, which we cannot examine, but which are all characterized

* Third edition. London, 1855.

† The crotchet figured in Ramsbotham's work on Obstetrics is, in our opinion, faulty, from the straightness of the shaft, but is far preferable to the one above alluded to.

‡ Lectures on Natural and Difficult Parturition.

by that great caution and sound judgment to which we have already alluded, and which make the work a reliable guide to the young and a useful companion to the old. There is one change which might be made in the arrangement of the work, however, which we believe would be an improvement. Were the cases in illustration of each variety of difficult labor placed immediately after the remarks upon the subject, as is done in that excellent work of the elder Ramsbotham, Practical Observations in Midwifery, we believe it would be perused with greater pleasure, if not more profit; such an arrangement serves to break that monotony which arises from reading several hundred cases one after the other. This, however, is a matter of minor importance; our chief regret in regard to the work is that the author has not given us more of it, both by extending his remarks upon the subjects treated of, and by including all within the limits of clinical obstetrics. We trust we may soon realize, what he intimates in his preface that we may hope for, further productions of his pen upon this subject.

